# Hippo pathway in cancer ferroptosis: from mechanism insights to targeted therapies

**DOI:** 10.3389/fmed.2026.1982771

**Published:** 2026-10-01

**Authors:** Ziyi Xu, Ruochen Yang, Hudabia Akram, Xinyu Gu

**Affiliations:** Henan Key Laboratory of Cancer Epigenetics, Cancer Institute, The First Affiliated Hospital, College of Clinical Medicine, Henan University of Science and Technology, Luoyang, Henan, China

**Keywords:** cancer therapy, drug resistance, ferroptosis, hippo pathway, YAP/TAZ

## Abstract

Ferroptosis is an iron-dependent form of regulated cell death driven by membrane lipid peroxidation and has become an important therapeutic vulnerability in cancer. The Hippo pathway, through its transcriptional effectors Yes-associated protein and transcriptional coactivator with PDZ-binding motif, links cell contact, mechanical stress, metabolism and tissue organization to cancer-cell state. Recent studies show that Hippo activity can either sensitize or protect tumor cells from ferroptosis, depending on the tissue microenvironment, effector-driven signaling and treatment pressure. This review integrates the molecular mechanisms of Hippo signaling and ferroptosis, then summarizes the evidence across four mechanistic layers: cell-contact and mechanotransduction inputs, direct control of ferroptosis machinery, metabolic and redox integration, and epigenetic or non-coding regulation. We further examine how this axis shapes cancer progression, stem-like states, drug resistance, tumor–stroma communication and immune therapy. Therapeutic strategies include direct disruption of YAP/TAZ–TEAD activity, pharmacological induction of ferroptosis, metabolic co-targeting, natural-product combinations, oncolytic viruses, engineered extracellular vesicles and nanomedicine. The major translational need is to define when Hippo activation marks ferroptosis vulnerability and when it marks ferroptosis resistance. Patient stratification, spatially resolved models, dynamic pharmacodynamic biomarkers and clinically realistic combination studies will be essential for translating these microenvironment-specific signaling cascades into a precision treatment strategy.

## 1 Introduction

Cancer therapy increasingly seeks vulnerabilities created by abnormal tumor metabolism rather than relying only on irreversible DNA damage. Ferroptosis has emerged as a particularly attractive form of regulated cell death because its execution depends on iron availability, polyunsaturated lipid oxidation and failure of antioxidant protection, processes that are frequently remodeled during malignant transformation. Accumulating evidence underscores ferroptosis as a critical tumor-suppressive mechanism and a viable therapeutic vulnerability, while recent work has emphasized its roles in tumor ecosystems and treatment design (1). The expanding research now connects ferroptosis to tumor suppression, stromal communication, immune regulation and pharmacological intervention (2). The field has therefore moved from defining ferroptosis toward identifying the cellular states that determine ferroptosis sensitivity (3).

This shift is clinically relevant because ferroptosis can be engaged by approved or investigational treatments, yet tumor cells may adapt through changes in cystine utilization, glutathione production, lipid remodeling and iron handling. Studies of cancer ferroptosis have highlighted the therapeutic promise of exploiting these dependencies and the need to match ferroptosis inducers with tumor-specific metabolic states (4). Prior investigations established the relevance of ferroptosis to cancer treatment and drug resistance, including the possibility of targeting ferroptosis in tumor cells that evade apoptosis (5, 6). Ferroptosis has consequently become linked to cancer stemness, therapy failure and combination treatment rather than being viewed as an isolated death phenotype (7).

The Hippo pathway provides a mechanistic bridge between tissue organization and ferroptosis. Its activity is shaped by cell density, cell–cell adhesion, extracellular matrix tension and oncogenic signaling, whereas its downstream effectors regulate transcriptional programs controlling proliferation, differentiation, metabolism and stress adaptation. The cystine transporter system Xc−, glutathione metabolism and ferroptosis-based reversal of drug resistance have become central themes in cancer research (8, 9). Ferroptosis also intersects with cancer immunotherapy and with malignancies such as colorectal and hepatocellular carcinoma (10–12).

YAP and TAZ are therefore positioned at a critical interface between the physical organization of a tumor and its biochemical response to therapy. The Hippo pathway is already being considered as a druggable cancer pathway, and YAP/TAZ biology has been connected to tumor progression, therapeutic response and clinical application (13–15). However, the relationship between Hippo activity and ferroptosis is not unidirectional. Depending on cancer type, cell state, metabolic background and therapeutic stress, YAP/TAZ activity may promote lipid peroxidation or reinforce antioxidant defense. This review summarizes how Hippo signaling determines ferroptosis sensitivity in cancer, integrates the major molecular mechanisms and biological consequences, and evaluates opportunities for biomarker development, resistance reversal and combination therapy.

## 2 Hippo signaling and ferroptosis in cancer

Hippo signaling and ferroptosis converge on a shared biological problem: how a cell translates changes in its physical and metabolic environment into a decision between survival and death. The Hippo pathway supplies the tissue-state component, whereas ferroptosis is executed through iron, lipid and redox metabolism. Their intersection is best understood as a condition-dependent network rather than as a simple linear pathway (Figure 1). Mechanistic characterization of Hippo–ferroptosis studies further supports the therapeutic importance of this interface in cancer (16).

**Figure 1:**
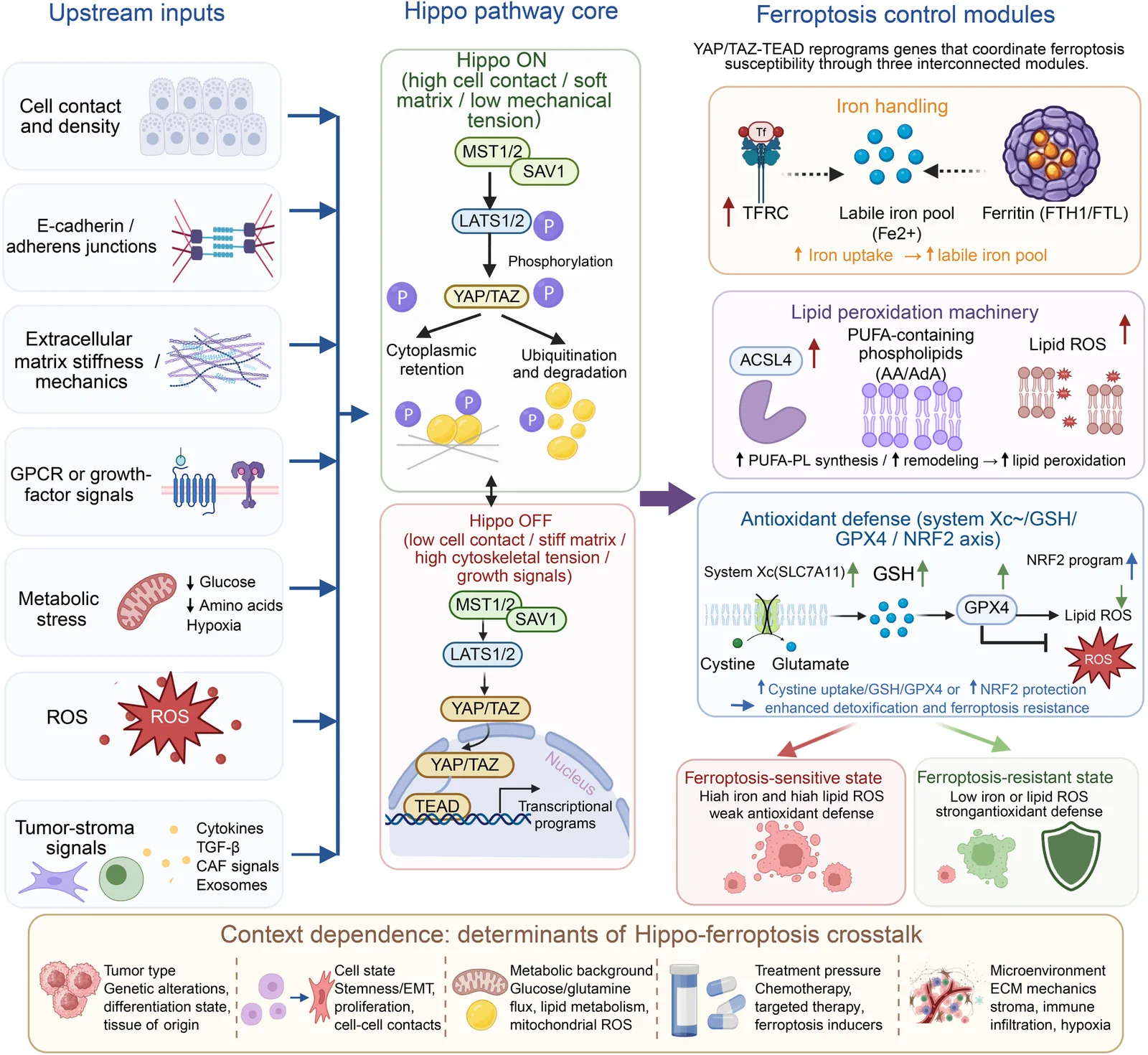
Hippo signaling and ferroptosis regulation in cancer. Cell contacts and density, E-cadherin-mediated adhesion, extracellular-matrix mechanics, GPCR or growth-factor signaling, metabolic stress, ROS, and tumor–stroma signals converge on the MST1/2–SAV1–LATS1/2 Hippo kinase cascade. Hippo activation promotes YAP/TAZ phosphorylation, cytoplasmic retention, and degradation, whereas Hippo inactivation allows nuclear YAP/TAZ–TEAD transcription. Downstream programs regulate ferroptosis through three major modules: iron handling via TFRC and the labile iron pool, lipid-peroxidation machinery involving ACSL4 and PUFA-containing phospholipids, and antioxidant defense mediated by system Xc−/SLC7A11, GSH, GPX4, and NRF2. The balance among these pathways determines ferroptosis-sensitive or ferroptosis-resistant states in a context-dependent manner.

### 2.1 Hippo pathway components and basic signaling

The canonical Hippo pathway contains the upstream tumor-suppressive kinase module MST1/2–SAV1 and the downstream kinase cascade LATS1/2, with NF2/Merlin and multiple membrane-associated regulators controlling signal input. When Hippo kinase activity is high, YAP and TAZ are phosphorylated, retained in the cytoplasm or targeted for degradation; when Hippo activity is low, YAP and TAZ enter the nucleus and cooperate mainly with TEAD transcription factors. This signaling axis was established as a regulator of organ size, tissue homeostasis and cancer growth (17). Comprehensive pathway analyses further showed that Hippo activity is regulated by upstream physical, receptor and metabolic cues rather than by a single dedicated ligand (18, 19).

YAP and TAZ are closely related transcriptional coactivators, but they are not interchangeable in every cancer model. Their nuclear activity is shaped by phosphorylation, protein stability, partner selection and chromatin state, and their downstream outputs can include growth, stemness, epithelial plasticity and metabolic adaptation (20). Interactions between YAP/TAZ and metabolism provide a direct functional link to ferroptosis because lipid synthesis, amino-acid utilization and redox buffering determine the availability of ferroptotic substrates and defenses (21). Mechanical inputs are particularly important because matrix stiffness, cell shape and force transmission alter YAP/TAZ localization and transcription (22).

In cancer, YAP/TAZ activity can be sustained by pathway mutation, loss of upstream tumor suppressors, altered adhesion, GPCR signaling or extracellular matrix remodeling. YAP/TAZ activation is rooted in cancer biology across tissues and is associated with oncogenic transcription, stem-cell behavior and therapeutic resistance (23). The pathway also contains canonical and non-canonical branches that operate in development, regeneration and disease (24). Liver-specific analyses further connect Hippo activity to tissue homeostasis, fibrosis and hepatocarcinogenesis, providing an important basis for HCC ferroptosis studies (25). These features make Hippo signaling an attractive therapeutic target, but they also predict that the effect of YAP/TAZ inhibition will depend on the tumor state and the stress imposed by treatment (26).

### 2.2 Ferroptosis pathways in cancer

Ferroptosis is characterized by iron-dependent accumulation of oxidized membrane lipids, with morphological and biochemical features that distinguish it from apoptosis, necroptosis and pyroptosis (27). The process is supported by a network of iron uptake, storage, lipid remodeling and antioxidant systems rather than by a single executioner protein (28, 29). Its core machinery includes iron trafficking, polyunsaturated fatty-acid incorporation, reactive oxygen species (ROS) generation and detoxification of lipid hydroperoxides (30).

The cystine–glutathione–glutathione peroxidase 4 axis is a major defense system. Cystine import through system Xc− supports glutathione synthesis, while glutathione peroxidase 4 (GPX4) reduces phospholipid hydroperoxides and prevents lethal membrane oxidation. Ferroptosis can also be restrained by ferritinophagy-dependent iron mobilization, mitochondrial pathways and alternative reducing systems (31, 32). The chemical biology of ferroptosis further shows that structurally distinct inducers and suppressors converge on lipid-peroxidation chemistry, but their activity is strongly influenced by cellular metabolism (33). Recent research integrates these molecular mechanisms with therapeutic strategies and reinforces the need for tumor-specific intervention (34).

Glutamine metabolism and transferrin-dependent iron uptake provide two examples of how nutrient flow controls ferroptosis. Glutaminolysis can support ferroptosis under defined conditions, whereas iron acquisition through transferrin increases the substrate available for oxidative injury (35). Autophagic degradation of ferritin can release iron and promote ferroptosis through ferritinophagy (36). Current cell-biological models also emphasize mitochondrial, organelle and metabolic contributions beyond the classical system Xc−–GPX4 axis (37).

Ferroptosis in cancer is therefore a balance between oxidation pressure and repair capacity. Regulatory networks involving p53, Nrf2, lipid metabolism, mitochondrial function and post-translational regulation can shift this balance (38). The same tumor can contain cells with different ferroptosis thresholds because of differences in density, differentiation, nutrient availability and interaction with stromal cells. This heterogeneity is essential for interpreting Hippo–ferroptosis studies.

### 2.3 The link between hippo activity and ferroptosis

The first direct connection between Hippo signaling and ferroptosis came from observations that cell density regulates ferroptosis sensitivity through YAP/TAZ-dependent transcription. YAP and TAZ were subsequently recognized as determinants that connect cell-state information to lipid, iron and ROS-regulatory genes (39, 40). A study proposed that this connection could be therapeutically exploited, especially in tumors with persistent YAP/TAZ activation (41).

YAP biology also intersects with several regulated cell-death programs, making the Hippo pathway a broader determinant of stress adaptation rather than a ferroptosis-specific switch (42). In cancer, YAP signaling has been linked to resistance to apoptosis and other treatment-induced death responses, which places ferroptosis within a wider drug-resistance network (43). Crosstalk with oncogenic signaling, lipid metabolism and post-translational regulation further expands the number of possible routes connecting Hippo activity to ferroptosis (44, 45).

The metabolic connection is especially important. Glutathione metabolism, lipid remodeling and ferroptosis defense are now viewed as therapeutic determinants, and the Hippo pathway can influence these processes through transcriptional and non-transcriptional mechanisms (46). This relationship explains why YAP/TAZ activation can have opposite effects in different tumors: activation of lipid-peroxidation programs may sensitize cells, whereas activation of cystine uptake or antioxidant programs may protect them.

### 2.4 Heterogeneity of hippo-ferroptosis crosstalk

Current evidence supports three broad patterns. In the first, Hippo effector activity promotes ferroptosis by increasing lipid oxidation or iron loading. In the second, YAP/TAZ activity protects tumor cells by reinforcing glutathione production, redox buffering or metabolic adaptation. In the third, Hippo signaling distributes ferroptosis sensitivity across neighboring cells and tumor compartments rather than determining death autonomously in a single cell. These patterns are consistent with the dual role of ferroptosis in tumor progression, cancer stemness and treatment resistance (47, 48).

The direction of the response can vary across prostate, breast, liver, lung and gastrointestinal tumors, and the same pathway may produce different outcomes under basal growth conditions and therapeutic stress. Studies in particular cancer types likewise show that ferroptosis biology is shaped by tissue metabolism and therapeutic regimens rather than by a uniform mechanism across all cancer (49, 50). Tumor microenvironment studies add a spatial dimension, indicating that immune, endothelial and stromal signals can change ferroptosis thresholds beyond the tumor cell itself (51). Hippo-dependent resistance to therapy should therefore be treated as a state-specific phenotype that requires experimental validation in models preserving tissue structure and treatment pressure (52).

## 3 Mechanisms linking hippo signaling to ferroptosis

Key studies converge on four interacting levels: upstream control by cell contact and mechanical state, direct transcriptional regulation of ferroptosis machinery, metabolic and redox integration, and epigenetic or non-coding modulation. These layers determine tumor growth, stem-like behavior, invasion and treatment response through the balance between lipid-peroxidation injury and ferroptosis defense (Figure 2; Table 1).

**Figure 2:**
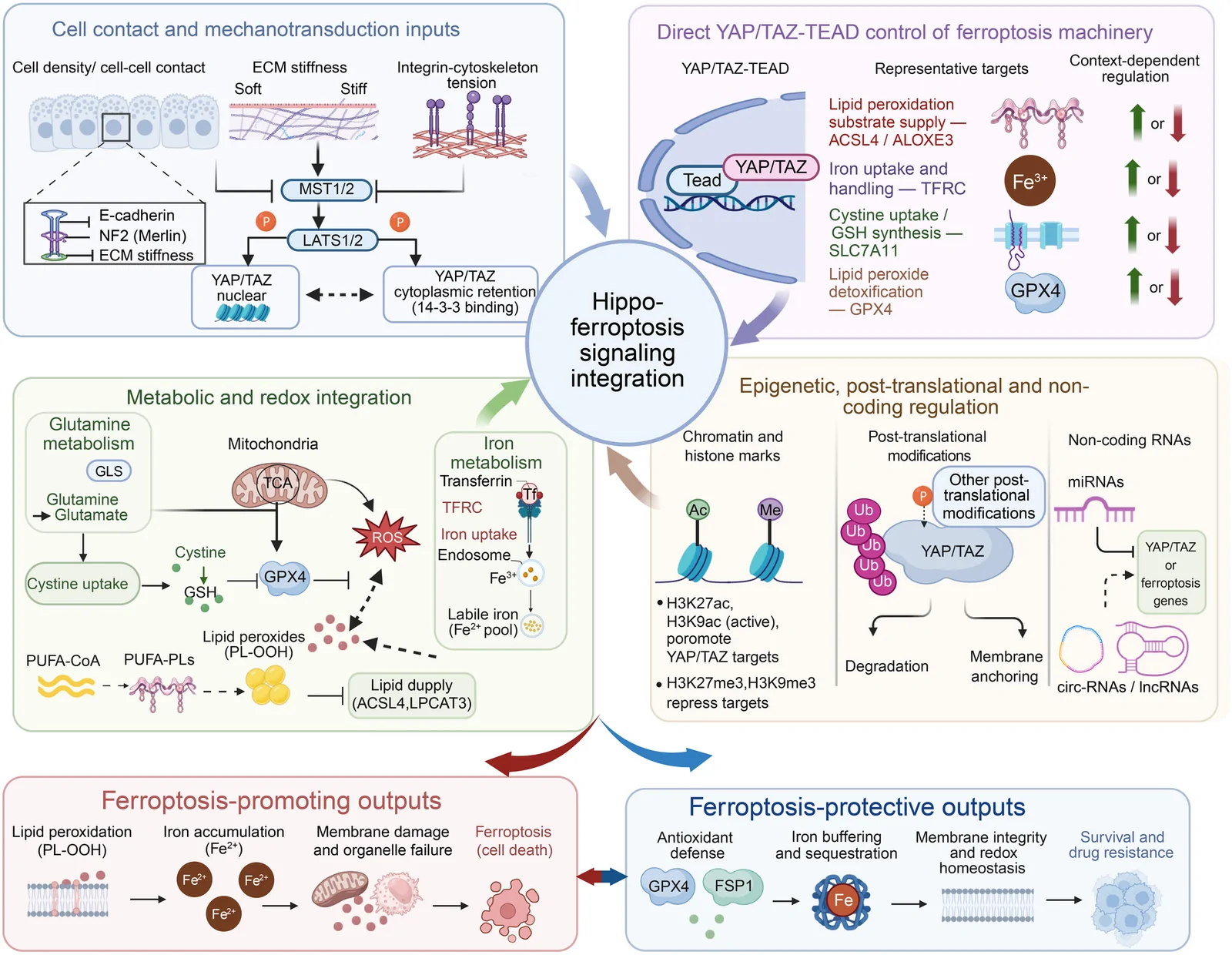
Molecular mechanisms linking hippo signaling to ferroptosis. The Hippo–ferroptosis axis is organized into four mechanistic layers: cell-contact and mechanotransduction inputs; direct YAP/TAZ–TEAD regulation of ferroptosis machinery; metabolic and redox integration; and epigenetic, post-translational, and non-coding regulation. Representative downstream targets include ACSL4/ALOXE3 for lipid-peroxidation substrate supply, TFRC for iron uptake, SLC7A11 for cystine uptake and GSH synthesis, and GPX4 for lipid-peroxide detoxification. These pathways converge on opposing ferroptosis-promoting and ferroptosis-protective outputs, thereby determining membrane damage, iron accumulation, antioxidant capacity, and cell survival.

**Table 1:** Core mechanisms linking hippo signaling to cancer ferroptosis.

Cancer type	Hippo regulator	Ferroptosis target/mechanism	Key finding	Evidence basis	Cell lines	Clinical translatability	REF.
Pan-cancer tumor cells	NF2/Merlin–YAP	Cell-contact-dependent ferroptosis	Cell interaction dictates ferroptosis through NF2–YAP signaling.	Cancer cells; interaction assays	MDA-MB-231; BT474; H1650; 211H; Meso33; CA-46; NF639	Preclinical; orthotopic *in vivo* validation; candidate predictive biomarker.	(53)
Renal cell carcinoma	TAZ	Density-dependent ferroptosis sensitivity	TAZ activity links cell density to ferroptosis susceptibility.	RCC cells; density assays	RCC4; 786O; 13-789; 293T	Preclinical; cell-based mechanistic evidence.	(54)
Epithelial ovarian cancer	TAZ	ANGPTL4–NOX2 regulation	TAZ–ANGPTL4–NOX2 signaling links confluence to ferroptosis.	Ovarian cancer cells; chemoresistance assays	CAOV2R; OVCA cell-line panel	Preclinical; cell-based mechanistic and chemoresistance evidence.	(55)
Recurrent breast cancer	DDR2–Hippo signaling	EMT-associated ferroptosis sensitivity	DDR2 increases ferroptosis susceptibility in recurrent tumors.	Mouse tumor cells; human tumor samples	SUM52; ZR751; BT474; MCF7; T47D; BT20; MDA-MB-231; Hs 578 T; BT549; MDA-MB-157	Preclinical; murine tumor-derived and human breast cancer cell-line evidence.	(56)
Meningioma	NF2/E-cadherin	Contact-dependent ferroptosis response	NF2 and E-cadherin status modifies erastin sensitivity.	Meningioma tissues; patient-derived cells	IOMM-Lee; CH157; patient-derived meningioma cells	Preclinical; human tumor specimens, patient-derived cells, and xenograft validation.	(57)
Diffuse gastric cancer	E-cadherin–Hippo	Cell adhesion and ferroptosis threshold	E-cadherin loss increases ferroptosis sensitivity.	Gastric cancer cells; genetic assays	SNU16; SNU668; 293T	Preclinical; xenograft validation; candidate predictive biomarker.	(58)
Oral squamous cell carcinoma	YAP/TAZ	Phase separation–ACSL4 regulation	Manganese activates YAP/TAZ phase separation and ferroptosis.	OSCC cells; patient specimens	SCC7	Candidate prognostic biomarker; supported by patient specimens and cell-based validation.	(59)
Breast carcinoma	YAP/TAZ–*β*4 integrin	Laminin 332–ZEB1 regulation	ECM signaling promotes ferroptosis resistance.	Breast cancer cells; mechanistic assays	BT549; HCC1806; HMT-3522 S1; TAZ4SA-S1	Preclinical; cell and patient-derived organoid evidence with human cohort-data support.	(60)
Breast cancer cells	YAP	YAP–SKP2 regulation	YAP promotes ferroptosis through the E3 ligase SKP2.	Cancer cells; genetic and chemical perturbation	MDA-MB-231; H1975; MCF7; T47D; HEK293 T; RCC4; CAOV2	Preclinical; cell-based mechanistic evidence.	(61)
Liver cancer	YAP	O-GlcNAcylation–YAP–TFRC	YAP stabilization increases TFRC and ferroptosis sensitivity.	HCC cells; iron and lipid assays	Bel-7402; SMMC-7721; HEK293T	Preclinical; xenograft validation.	(62)
Hepatocellular carcinoma	YAP	ALOXE3-mediated lipid peroxidation	YAP activity sensitizes HCC to ferroptosis through ALOXE3.	HCC tissues; cell and tumor analyses	LM3; HuH-7; SK-HEP-1; HepG2; Hepa1-6; HEK-293T	Preclinical; human HCC tissue and tumor-model support; candidate predictive biomarker.	(63)
Breast cancer	SUFU–YAP	YAP–ACSL4 regulation	SUFU depletion activates YAP and increases ACSL4-dependent ferroptosis.	Breast cancer cells; genetic perturbation	MDA-MB-231; HCC1954; MCF-7; SK-BR-3; T47D	Preclinical therapeutic strategy; *in vitro* and *in vivo* validation.	(64)
Clear cell renal cell carcinoma	TAZ	TAZ–SLC7A11 defense	MITD1 loss induces ferroptosis through TAZ–SLC7A11 regulation.	ccRCC cells; knockdown experiments	HK-2; 786-O; ACHN; A498; 769-P; Caki-1	Preclinical; bioinformatic and cell-based mechanistic evidence.	(65)
Non-small cell lung cancer	HIF-1*α*–Hippo–YAP	YAP-dependent ferroptosis suppression	HIF-1α activates Hippo–YAP signaling and inhibits ferroptosis.	NSCLC cells; genetic and pharmacological assays	SW900; A549; BEAS-2B	Preclinical; cell-based mechanistic evidence.	(66)
Hepatocellular carcinoma	AKR1C3–YAP	YAP–SLC7A11 defense	AKR1C3 depletion sensitizes HCC to ferroptosis.	HCC cells; mouse model	/	Preclinical; *in vitro* and *in vivo* validation with prognostic association.	(106)
Glioma	CMTM5–WWP2–LATS2	Hippo activation and ferroptosis	CMTM5 promotes ferroptosis through LATS2 regulation.	Glioma cells; nude-mouse model	A172; LN229; HEK293T	Preclinical; glioma cell and nude-mouse validation.	(67)
Prostate cancer	CYLD–YAP	YAP ubiquitination and ferroptosis	CYLD enhances ferroptosis by restraining YAP signaling.	PCa cells; mouse model	DU145; LNCaP; PC-3; 22RV1; RWPE-1; C4-2	Preclinical; prostate cancer cell and mouse-model validation.	(68)
Non-small cell lung cancer	MISP–MST1/2–YAP	Hippo kinase inhibition	MISP activates YAP and suppresses ferroptosis.	NSCLC cells; mouse model	HEK293 T; PC-9; NCI-H1395; NCI-H1975; HCC827; BEAS-2B; 16HBE	Preclinical; NSCLC tissue and cell evidence with *in vivo* validation.	(69)
Bladder cancer	TAZ–NRF2	Antioxidant feedback	TAZ–NRF2 feedback promotes growth and ferroptosis resistance.	Bladder cancer cells; mouse model	T24; 5637; BIU-87; UM-UC-3; EJ; J82; SV-HUC-1	Preclinical; tumor-tissue, cell, and *in vivo* validation.	(70)
Esophageal squamous cell carcinoma	CDK7–YAP–LDHD	D-lactate metabolism	CDK7–YAP–LDHD supports ferroptosis defense in cancer stem-like cells.	ESCC cells; stem-like tumor analyses	/	Preclinical; patient-tissue and xenograft validation with prognostic association.	(71)
Castration-resistant prostate cancer	YAP1	SLC1A5–GLS1–GSH metabolism	YAP1 drives glutamine metabolism and suppresses ferroptosis.	CRPC tissues; inhibitor-treated cells	PC-3; DU145	Preclinical; CRPC tissue and cell-based metabolic evidence.	(72)
Clear cell renal cell carcinoma	ENO2–Hippo–YAP1	Glycolysis and ferroptosis	ENO2 interference promotes ferroptosis and inhibits glycolysis.	ccRCC cells; metabolic assays	HK-2; 786-O; ACHN; Caki-1; 769-PRCC	Preclinical; cell-based mechanistic evidence.	(107)
Breast cancer	YAP–DDX3X	Lipid-peroxidation suppression	YAP-mediated DDX3X reduces lipid peroxidation and resistance.	Breast cancer cells; mechanistic assays	MCF7; MDA-MB-231; BT549; T47D; HCC1806	Preclinical; cell-based mechanistic evidence.	(73)
Colorectal cancer	SLC38A5–Hippo–YAP/Nrf2	Nutrient transport and antioxidant defense	SLC38A5 drives ferroptosis resistance through YAP/Nrf2.	CRC cells; validation studies	DLD1; HCT116	Preclinical; cell-based mechanistic evidence.	(74)
Prostate cancer	YAP/TEAD4–BRD9–DGAT1	Lipid-droplet deposition	BRD9–DGAT1 promotes ferroptosis resistance in YAP-high tumors.	PCa cells; orthotopic mouse model	22RV-1; 293 T; C4-2	Preclinical therapeutic target; orthotopic *in vivo* validation.	(75)
Breast cancer	YAP–GCH1	Cofactor-dependent ferroptosis vulnerability	YAP induces ferroptosis vulnerability through GCH1 suppression.	Breast cancer cohorts; cell models	4T1	Preclinical; patient-cohort association and murine cell-model validation.	(76)
Lung cancer	TBX5–YAP1–TEAD1	Transcriptional ferroptosis control	TBX5 suppresses YAP1–TEAD1 and promotes ferroptosis.	Lung cancer cells; genetic perturbation	A549; NCI-H1703	Preclinical; cell-based mechanistic evidence.	(77)
Esophageal squamous cell carcinoma	IQGAP1–YAP	Ferroptosis inhibition and paclitaxel response	IQGAP1 promotes YAP-mediated ferroptosis inhibition.	ESCC tissues; cell models	/	Preclinical; human ESCC tissue association and cell-based chemosensitivity evidence.	(78)
Non-small cell lung cancer	circPOLA2–Merlin–YAP	Non-coding RNA regulation	circPOLA2 activates Merlin–YAP signaling and ferroptosis.	NSCLC tissues; cell and tumor models	A549; PC9	Preclinical; NSCLC tissue, cell, and tumor-model validation.	(79)
Epithelial ovarian cancer	MED12–YAP–TEAD1	Ferroptosis sensitivity control	MED12 deficiency increases ferroptosis sensitivity.	EOC cells; CRISPR/rescue assays	SKOV3; HO8910	Preclinical; cell-based CRISPR/rescue mechanistic evidence.	(80)
Meningioma	KMT2C–NF2–Hippo	Epigenetic pathway repression	KMT2C loss alters NF2 and Hippo-dependent ferroptosis.	Meningioma tissues; cell and *in vivo* models	IOMM-Lee; CH157MN	Preclinical; meningioma tissue, cell, and *in vivo* validation; candidate therapeutic target.	(81)

### 3.1 Cell contact, density and mechanotransduction

Cell contact is an upstream gatekeeper of the Hippo–ferroptosis axis because adhesion proteins and cytoskeletal tension simultaneously regulate tissue organization and oxidative stress. In cancer cells, interaction between neighboring cells can engage NF2/Merlin–YAP signaling and determine whether a ferroptotic signal is transmitted across the tumor-cell population (53). In renal cell carcinoma (RCC), ferroptosis sensitivity changes with cell density, while TAZ localization and activity shift between sparse and confluent states (54).

The same density principle extends to epithelial ovarian cancer, where TAZ links cell confluence to the ANGPTL4–NOX2 axis and chemoresistance (55). These findings establish cell density as a metabolic variable rather than a mere culture artifact. They also suggest that the ferroptosis phenotype of a tumor cell is partly determined by the cellular neighborhood in which that cell resides.

Extracellular matrix and adhesion signals further refine this gate. In recurrent breast tumors, epithelial–mesenchymal transition regulators increase DDR2 and enhance ferroptosis susceptibility through a Hippo-related program (56). In meningioma, NF2 and E-cadherin status associates with ferroptosis response, and loss of these contact-related regulators alters sensitivity to erastin (57). In diffuse gastric cancer, loss of E-cadherin increases ferroptosis sensitivity, whereas homophilic E-cadherin contacts activate Hippo signaling and suppress ferroptotic death (58).

Mechanical and phase-separation mechanisms add another layer of control. Manganese-induced ferroptosis in oral squamous cell carcinoma involves YAP/TAZ phase separation and ACSL4 activation, indicating that the physical organization of transcriptional regulators can influence lipid-peroxidation programs (59). In breast carcinoma, the *β*4 integrin–laminin 332 axis sustains YAP/TAZ-dependent epithelial programs and promotes ferroptosis resistance through repression of ZEB1 (60). Together, these studies place mechanical and adhesive inputs upstream of the iron–lipid–redox machinery, but they also expose a limitation: density, substrate stiffness and cell composition are rarely standardized across models. The apparent direction of YAP/TAZ action may therefore depend on whether the experimental system preserves the native tissue structure of the tumor.

### 3.2 YAP/TAZ–TEAD control of ferroptosis machinery

YAP and TAZ can act directly on ferroptosis through gene expression programs that regulate lipid oxidation and iron handling. YAP removal confers ferroptosis resistance, whereas YAP overexpression sensitizes cancer cells, with SKP2 identified as a direct YAP target required for lipid peroxidation (61). In liver cancer, O-GlcNAcylation stabilizes YAP, increases TFRC transcription and raises intracellular iron, thereby enhancing sensitivity to RSL3-induced ferroptosis (62).

YAP activity can also promote ferroptosis through lipid-peroxidation enzymes. In hepatocellular carcinoma (HCC), oncogenic YAP signaling increases ALOXE3-mediated lipid peroxidation and provides a potential marker of response to ferroptosis-inducing therapy (63). In breast cancer, depletion of SUFU activates YAP and increases ACSL4-dependent ferroptosis, linking Hedgehog-associated regulation to the Hippo pathway and membrane lipid remodeling (64). These findings support a vulnerability model in which high YAP activity can be advantageous for tumor growth while simultaneously creating dependence on ferroptotic lipid chemistry.

The opposite direction is evident when YAP/TAZ activate antioxidant defense. In clear cell RCC, MITD1 deficiency induces ferroptosis through the TAZ–SLC7A11 pathway and suppresses tumor growth, indicating that TAZ activity can protect cells from ferroptotic stress (65). In non-small cell lung cancer (NSCLC), hypoxia-inducible factor 1*α* suppresses ferroptosis while activating the Hippo–YAP axis, and the phenotype is partially reversed by ferroptosis modulators (66). In HCC, AKR1C3 knockdown increases ferroptosis sensitivity through the YAP/SLC7A11 pathway in cell and animal models (66).

Hippo kinase regulation can reinforce this protective state. CMTM5 promotes ferroptosis in glioma by regulating WWP2-mediated LATS2 ubiquitination and the Hippo–YAP axis (67). In prostate cancer, CYLD enhances ferroptosis *in vitro* and *in vivo* by restraining YAP ubiquitination and signaling, providing a complementary example of deubiquitinase control (68). MISP suppresses ferroptosis in NSCLC by inhibiting MST1/2 kinase activity and facilitating YAP activation, with *in vivo* evidence for reduced tumor growth after MISP loss (69). TAZ also forms a positive feedback loop with NRF2 in bladder cancer, supporting proliferation while antagonizing ferroptosis (70). These results indicate that YAP/TAZ cannot be classified as universally pro-ferroptotic or anti-ferroptotic; the decisive variable is the downstream transcriptional program engaged in a specific tumor state.

### 3.3 Metabolic and redox integration

Hippo signaling regulates ferroptosis partly by reorganizing how tumor cells obtain reducing equivalents and manage oxidized lipids. In esophageal squamous cell carcinoma (ESCC), CDK7 phosphorylation enhances YAP transcriptional activity and increases LDHD, supporting D-lactate elimination and ferroptosis defense in cancer stem-like cells (71). In castration-resistant prostate cancer (CRPC), YAP1 drives glutamine uptake and metabolism through SLC1A5 and GLS1, increasing glutathione production and inhibiting RSL3-induced ferroptosis (72).

Glycolytic and amino-acid programs can converge on the same axis. ENO2 interference promotes ferroptosis and suppresses glycolysis in clear cell RCC through Hippo–YAP1 signaling (72). In breast cancer, YAP-mediated DDX3X activity reduces lipid peroxidation and confers ferroptosis resistance, linking RNA metabolism to the redox state of tumor cells (73). SLC38A5 drives colorectal cancer ferroptosis resistance through a Hippo–YAP/Nrf2 axis, providing a direct connection between nutrient transport, antioxidant transcription and tumor progression (74).

Lipid-storage and cofactor pathways further expand the metabolic network. In YAP-high prostate cancer, a BRD9–DGAT1 axis promotes lipid-droplet deposition and ferroptosis resistance, suggesting that YAP-driven epigenetic programs can alter the availability of oxidizable membrane lipids (75). Conversely, YAP suppresses GCH1 in breast cancer and thereby increases ferroptosis vulnerability, illustrating that YAP activity may remove a protective cofactor pathway in one setting while activating antioxidant defenses in another (76). The biological meaning of YAP activation therefore depends on whether its downstream program increases oxidizable substrates, iron loading or antioxidant capacity.

### 3.4 Epigenetic, post-translational and non-coding regulation

Upstream regulators modify the Hippo–ferroptosis axis through changes in transcription-factor activity, protein stability and chromatin state. TBX5 inhibits the YAP1–TEAD1 pathway and promotes ferroptosis in lung cancer cells, indicating that transcription factors outside the canonical Hippo core can determine ferroptotic response (77). IQGAP1 overexpression attenuates chemosensitivity in ESCC by inhibiting YAP-mediated ferroptosis, linking a scaffold protein to both drug response and ferroptosis regulation (78).

Non-coding RNA and extracellular communication can also influence Hippo activity. CircPOLA2 sensitizes NSCLC cells to ferroptosis and suppresses tumorigenesis through the Merlin–YAP pathway, connecting RNA regulation to the upstream contact-control module (79). In epithelial ovarian cancer, MED12 controls ferroptosis sensitivity through YAP–TEAD1 signaling, with CRISPR and rescue experiments supporting a causal role for the MED12–Hippo interface (80). In NF2-wild-type meningioma, KMT2C loss represses NF2 transcription and changes Hippo signaling while increasing tumor aggressiveness and ferroptosis sensitivity (81).

These findings show that Hippo–ferroptosis regulation is not limited to transcriptional activity from nuclear YAP/TAZ. It also depends on protein modification, chromatin accessibility, RNA localization and the ability of cells to preserve or lose tissue-state information. This multilayered regulation helps explain why single-gene biomarkers often perform inconsistently across cancer types and why combination strategies may be more robust than isolated pathway inhibition.

### 3.5 Biological consequences in different cancers

The mechanistic studies collectively support a model in which Hippo signaling controls more than cell death. It influences whether tumor cells remain proliferative, acquire stem-like properties, undergo epithelial–mesenchymal transition, survive treatment or compete with neighboring cells. Ferroptosis sensitivity can therefore function as a biological consequence of a broader cell-state program rather than as an isolated endpoint.

Across tumors, three recurring outcomes are evident. First, high YAP/TAZ activity can create ferroptotic vulnerability through lipid-peroxidation or iron-loading programs. Second, the same activity can support ferroptosis resistance through cystine uptake, glutathione production, Nrf2 activation or lipid storage. Third, Hippo heterogeneity can partition a tumor into rapidly growing but ferroptosis-sensitive cells and slower but ferroptosis-resistant cells. These outcomes argue against a universal therapeutic rule and support tumor-specific biomarker development.

Several representative studies illustrate that Hippo modulation can produce substantial changes in ferroptosis susceptibility rather than modest biochemical shifts. In the renal cell carcinoma study summarized in Table 1, RCC4 cells grown at low density (<50% confluence) were highly sensitive to erastin, whereas high-density cells (>80% confluence) showed more than a two-fold increase in erastin EC50. TAZ depletion reproduced the ferroptosis-resistant phenotype associated with high cell density, whereas constitutively active TAZ markedly increased ferroptosis sensitivity. Similarly, the hepatocellular carcinoma sorafenib-resistance study summarized in Table 2 showed that disruption of YAP/TAZ-dependent antioxidant defense substantially enhanced sorafenib-induced ferroptosis and suppressed xenograft tumor growth. Pharmacological inhibition of the SLC7A11–GSH defense pathway also delayed tumor growth and increased lipid-peroxidation markers *in vivo*. Together, these findings indicate that Hippo-dependent regulation can shift ferroptosis sensitivity sufficiently to affect both cancer-cell survival and therapeutic response. Nevertheless, direct comparison of effect sizes across studies remains difficult because the experimental models, ferroptosis inducers, doses and endpoints are heterogeneous.

**Table 2:** Biomarker and therapeutic evidence for the hippo–ferroptosis axis.

Cancer context	Intervention or marker	Hippo–ferroptosis mechanism	Translational outcome	Evidence basis	Cell lines	Clinical translatability	REF.
Ovarian clear cell carcinoma	ZDHHC7/YAP1 status	YAP1 suppression and ferroptosis resistance	Candidate marker of resistance and poor prognosis.	OCCC tissues; cell models	RMG-I; 17 ovarian cancer cell lines (screening panel)	Candidate biomarker; human OCCC tissue association with preclinical cell validation.	(82)
Hepatocellular carcinoma	Sorafenib plus Hippo disruption	YAP/TAZ–ATF4–SLC7A11 defense	Hippo disruption restores sorafenib-induced ferroptosis.	HCC cells; xenograft model	HEK-293 T; SNU398; HT1080; Huh7; HLE; Hep3B; Platinium-A	Preclinical therapeutic strategy; patient-tissue support and xenograft validation.	(83)
Head and neck cancer	Gefitinib response	EMP1-linked ferroptosis regulation	Connects ferroptosis sensitivity with gefitinib resistance.	HNSCC cells; drug-response assays	Hep2; Detroit562; TU686; NP69-SV40T	Preclinical; cell-based drug-resistance evidence with therapeutic-target potential.	(84)
Pancreatic cancer	Gemcitabine response	PDK4–Hippo-mediated ferroptosis inhibition	Defines a ferroptosis-linked gemcitabine resistance mechanism.	PAAD tissues; cell models	ASPC1	Preclinical; gemcitabine-resistant patient-tissue association and cell validation.	(85)
Esophageal cancer	Cisplatin response	CAF Tenascin-C–Hippo/TAZ signaling	Identifies stromal control of ferroptosis and resistance.	ESCC tissues; cell models	TE-1; KYSE450	Preclinical; human tissue and experimental-model support; candidate biomarker and therapeutic target.	(86)
Ovarian cancer	Cisplatin response	RUNX2 palmitoylation–YAP1/GLS1	Links post-translational regulation to chemoresistance.	Ovarian cancer cells; xenografts	OVCAR3; A2780; HO8910; IOSE80; SKOV3; A2780/CDDP; SKOV3/CDDP	Preclinical chemoresistance-targeting strategy; xenograft validation.	(108)
Ewing sarcoma	AURKA inhibitor	NPM1/YAP1-dependent ferroptosis	Engages ferroptosis and apoptosis through kinase inhibition.	Ewing sarcoma cells; tumor models	A673; SKNMC; RDES; SK-NEP-1; BMSC	Preclinical therapeutic strategy; xenograft validation with prognostic association.	(87)
Tumor cells	Newcastle disease virus	PRKN-mediated YAP degradation	Provides a virus-based route to enhance ferroptosis.	Tumor cells; oncolytic-virus model	HeLa; H1299; A549; HepG2; DF-1	Preclinical oncolytic-virus proof-of-concept; tumor-cell mechanistic evidence.	(88)
Gastric cancer	Periplocymarin	ATP1A1–Src–YAP/TAZ–TFRC	Sensitizes gastric cancer cells to ferroptosis.	Gastric cancer cells; functional assays	HGC27; MKN45; AGS; FU97; HT29; GES1; HEK293T	Preclinical combination strategy; xenograft efficacy with preliminary organ-safety assessment.	(89)
Non-small cell lung cancer	Schisantherin A	YAP–ACSL4–transferrin-receptor signaling	Induces ferroptosis through coordinated iron-lipid regulation.	NSCLC cells; functional assays	HCC827; A549	Preclinical natural-product strategy; nude-mouse xenograft validation.	(90)
Breast cancer	Ginkgetin plus paclitaxel	MDM2–p53–YAP1 ferroptosis signaling	Enhances paclitaxel-associated ferroptosis.	Breast cancer cells; combination assay	MCF-7	Preclinical combination strategy; cell line-derived xenograft validation.	(91)
Hepatocellular carcinoma	SAMD1 targeting plus anti-PD-1/lenvatinib	Increased ferroptosis and immune response	Enhances combined immunotherapy and targeted therapy.	HCC cells; *in vivo* therapy	Huh7; Hep3B	Preclinical immunotherapy-combination strategy; *in vivo* nanoparticle-delivery validation.	(93)
Melanoma	Engineered miR-150-3p nanovesicles	YAP-dependent ferroptosis and T-cell remodeling	Combines ferroptosis induction with immune reprogramming.	Melanoma model; nanovesicles	PIG1; A375; A875; B16-F10; HEK293T	Preclinical nanovesicle/immunotherapy strategy; *in vivo* melanoma-model validation.	(94)
Pancreatic cancer	Copper-verteporfin plus IKE	YAP inhibition and ferroptosis induction	Reverses SLC7A11/GPX4-linked ferroptosis resistance.	Pancreatic cancer model; nanoparticles	/	Preclinical nanomedicine strategy; *in vivo* tumor-growth inhibition.	(95)
Breast cancer	YAP1-inhibitory LBP nanoparticles	YAP1 suppression and metabolic remodeling	Induces ferroptosis and inhibits tumor progression.	Breast cancer cells; nanoparticle treatment	MCF10A; MDA-MB-231	Early preclinical nanomedicine proof-of-concept; *in vitro* validation only.	(96)
Gastric cancer	Endothelial-cell exosomal SLC1A5	EGFR/SRC/YAP1/GPX4 activation	Demonstrates stromal exosome-mediated ferroptosis resistance.	Gastric cancer cells; exosome model	HUVECs; MGC803; HGC27; 293T	Preclinical tumor-microenvironment target; xenograft validation.	(97)
Lung cancer	Hippo-regulated biopterin metabolism	Non-cell-autonomous ferroptosis protection	Identifies a metabolic shield between tumor-cell states.	Lung adenocarcinoma; human and murine models	LLC; LK-2; PC-9; H1975; A549	Preclinical; human lung adenocarcinoma data with murine-model validation.	(98)
Colorectal cancer	USP52 inhibition	YAP stabilization and ferroptosis suppression	Links Hippo-dependent ferroptosis resistance to immunotherapy failure.	CRC cells; treatment-response assays	SW480; HCT116; MC38; HEK293	Preclinical immunotherapy-sensitization target; *in vivo* validation.	(99)
Lung cancer	Hippo pathway inhibition	Durable non-cell-autonomous resistance	Highlights spatially maintained ferroptosis-resistant niches.	Lung cancer; tumor-cell interaction models	LLC; A549; LK-2; PC9; H1975	Preclinical spatial resistance mechanism; murine *in vivo* validation.	(100)

## 4 Clinical and therapeutic implications

The translational value of the Hippo–ferroptosis axis lies in linking a measurable tumor state to a modifiable treatment vulnerability. Current evidence spans response prediction, drug resistance, direct pathway targeting, immune and stromal interactions, and nanomedicine. These relationships are summarized in the translational pathway and evidence matrix (Figure 3; Table 2).

**Figure 3:**
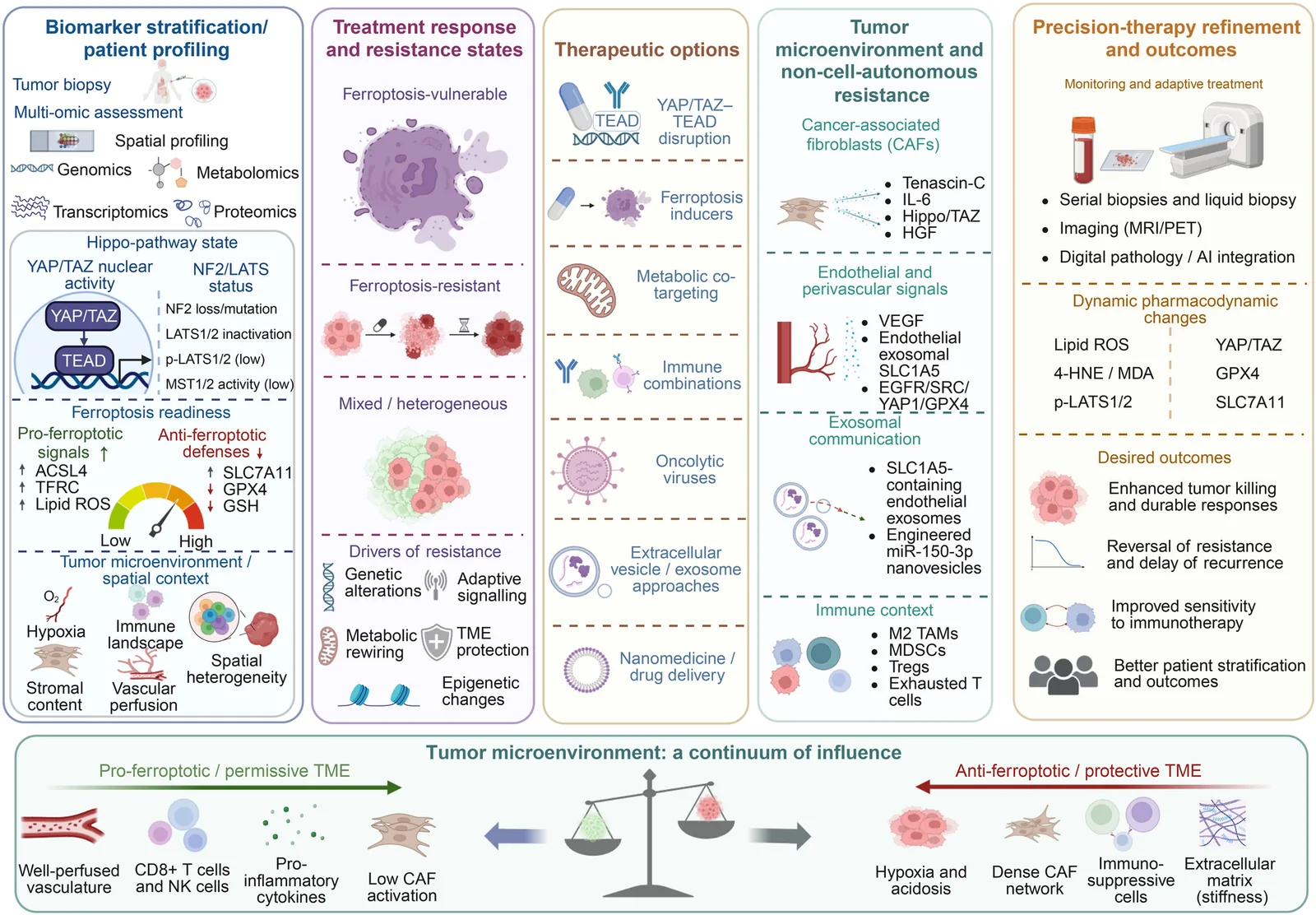
Translational targeting of the hippo–ferroptosis axis. Biomarker-based patient stratification integrates Hippo-pathway activity, ferroptosis readiness, and tumor-microenvironmental context to identify ferroptosis-vulnerable, resistant, or heterogeneous tumor states. Therapeutic strategies include YAP/TAZ–TEAD disruption, ferroptosis inducers, metabolic co-targeting, immune combinations, oncolytic viruses, extracellular-vesicle approaches, and nanomedicine. Tumor-microenvironmental factors, including CAF-derived Tenascin-C, endothelial exosomal SLC1A5 signaling, engineered miR-150-3p nanovesicles, and immune-cell interactions, can influence non-cell-autonomous ferroptosis resistance. Dynamic pharmacodynamic monitoring of lipid ROS, 4-HNE/MDA, p-LATS1/2, YAP/TAZ, GPX4, and SLC7A11 may support treatment adaptation and patient stratification.

### 4.1 Biomarkers and patient stratification

The most immediate clinical question is whether Hippo activity can identify patients whose tumors are susceptible to ferroptosis-inducing therapy. In ovarian clear cell carcinoma (OCCC), suppression of YAP1 by ZDHHC7 associates with ferroptosis resistance and poor prognosis, supporting YAP1 status as a candidate stratification feature (82). This finding is particularly relevant to OCCC because oxidative stress and iron accumulation are intrinsic features of its disease biology, but the evidence still supports a candidate biomarker rather than a clinically validated test.

Potential biomarker panels should combine pathway activity with ferroptosis execution and defense markers. Nuclear YAP/TAZ, E-cadherin or NF2 status, ACSL4, TFRC, SLC7A11, GPX4 and lipid-peroxidation signatures may provide more informative stratification than any single protein. Such panels should distinguish prognostic association from predictive value and should be measured dynamically during treatment rather than only in pretreatment tissue.

### 4.2 Therapy response and resistance

HCC provides the clearest example of Hippo-mediated treatment resistance. YAP/TAZ and ATF4 promote resistance to sorafenib by maintaining SLC7A11-dependent antioxidant protection and preventing drug-induced ferroptosis, while combined pathway disruption and sorafenib suppress tumor growth in xenografts (83). The same logic extends to other treatment settings. EMP1-associated signaling links ferroptosis regulation to gefitinib resistance in head and neck cancer (84). PDK4 activates Hippo signaling, inhibits ferroptosis and reduces gemcitabine sensitivity in pancreatic cancer (85).

Stromal signaling can impose treatment resistance from outside the tumor cell. Cancer-associated fibroblast-derived Tenascin-C activates Hippo signaling, suppresses TAZ, reduces ferroptosis and confers cisplatin resistance in ESCC (86). In ovarian cancer, ZDHHC14-driven RUNX2 palmitoylation attenuates ferroptosis and enhances chemoresistance through a YAP1/GLS1 axis (86). These studies indicate that resistance is not only a tumor-cell-autonomous transcriptional event; it can be sustained by stromal signals and post-translational control.

### 4.3 Therapeutic targeting and combination strategies

Therapeutic approaches can target the pathway via multiple pharmacological nodes. AURKA inhibition induces apoptosis and ferroptosis through the NPM1/YAP1 axis in Ewing sarcoma, illustrating how upstream kinase inhibition can engage multiple death programs (87). Oncolytic Newcastle disease virus promotes YAP degradation through PRKN and enhances ferroptosis in tumor cells, providing a biological delivery route that combines infection with pathway disruption (88).

Natural products represent a mechanistically distinct class of ferroptosis modulators within the Hippo network. Compared with synthetic ferroptosis inducers that often act on defined nodes such as system Xc− or GPX4, natural compounds may provide structurally diverse and polypharmacological scaffolds capable of simultaneously modifying upstream signaling, iron handling, lipid metabolism and redox defense. For example, the cardiac glycoside periplocymarin targets ATP1A1 and activates Src, which promotes YAP/TAZ tyrosine phosphorylation and nuclear translocation independently of the canonical Hippo kinase cascade. The resulting YAP/TAZ–TEAD complex directly enhances TFRC transcription, increasing iron uptake and lipid peroxidation and thereby sensitizing gastric cancer cells to ferroptosis (89). Schisantherin A similarly increases YAP activity in NSCLC, leading to upregulation of ACSL4 and transferrin receptor, increased intracellular Fe^2^⁺ accumulation and depletion of glutathione, ultimately promoting ferroptotic death (90). Ginkgetin acts through a different regulatory route: inhibition of the MDM2–p53 interaction promotes p53 activity and YAP1 signaling while reducing GPX4, SLC7A11 and related antioxidant defenses, thereby increasing ferroptosis and enhancing the antitumor activity of paclitaxel in breast cancer (91). These examples suggest that the potential advantage of natural compounds lies less in intrinsic superiority over synthetic agents than in their ability to engage multiple ferroptosis-relevant nodes and to function as sensitizers in combination therapy. Synthetic ferroptosis inducers generally offer greater target definition and experimental controllability, which facilitates pharmacodynamic interpretation and structure–activity optimization. In contrast, natural products frequently exhibit broader target spectra and may simultaneously affect several ferroptosis-regulatory pathways, potentially making them useful as combination sensitizers or lead scaffolds for drug development. However, such polypharmacology can also complicate target attribution, dose optimization and prediction of off-target effects. Therefore, current evidence does not establish a general therapeutic superiority of natural over synthetic ferroptosis modulators (92).

Combination delivery may improve pathway selectivity. Targeting SAMD1 enhances anti-PD-1 plus lenvatinib treatment in HCC by increasing ferroptosis sensitivity and immune response (93). Engineered nanovesicles can trigger YAP-dependent ferroptosis while delivering miR-150–3p to remodel T-cell immunity in melanoma (94). Copper-verteporfin coordination nanoparticles combine YAP inhibition with a ferroptosis inducer to reverse pancreatic cancer resistance associated with SLC7A11 and GPX4 (95). Lipid-based nanoparticles that inhibit YAP1 can also induce ferroptosis and alter breast cancer metabolism (96).

These strategies share a central principle: Hippo modulation is most useful when paired with a defined ferroptosis-inducing pressure. Direct YAP/TAZ inhibition may be insufficient if iron loading or lipid oxidation remains limiting, whereas ferroptosis induction may fail if YAP/TAZ-dependent antioxidant programs are not removed.

### 4.4 Tumor microenvironment and non-cell-autonomous ferroptosis

Tumor microenvironmental communication expands the therapeutic scope of Hippo–ferroptosis biology. Senescent endothelial-cell-derived exosomal SLC1A5 activates EGFR/SRC/YAP1/GPX4 signaling in gastric cancer and suppresses ferroptosis, linking obesity-associated vascular signals to tumor progression (97). In lung cancer, heterogeneous YAP/TAZ activity creates distinct tumor-cell states, and Hippo-regulated biopterin metabolism can shield neighboring cells from ferroptosis (98).

These non-cell-autonomous effects are relevant to immunotherapy. USP52 stabilizes YAP, inhibits ferroptosis and blocks immunotherapy response in colorectal cancer, indicating that Hippo-dependent ferroptosis resistance can influence treatment beyond direct cytotoxicity (99). A further lung-cancer study described durable non-cell-autonomous ferroptosis resistance driven by Hippo signaling, reinforcing the idea that spatial organization and cell–cell communication can preserve resistant niches (100).

The therapeutic implication is that tumor-cell-only assays may underestimate resistance. Co-culture models, organoids, spatial profiling and immune-competent animal models are needed to determine whether a treatment eliminates ferroptosis-resistant neighbors or merely selects them.

### 4.5 Translational barriers and clinical development priorities

The first barrier is biological directionality. YAP/TAZ activation may promote or suppress ferroptosis depending on the downstream program, and pathway activity cannot be interpreted without measuring iron, lipid-peroxidation and antioxidant outputs in the same tumor. The second barrier is model dependence. Cell density, matrix composition, oxygen tension, nutrient availability and stromal composition can all change Hippo activity and ferroptosis thresholds.

The third barrier is biomarker standardization. Nuclear YAP, total YAP, TAZ, SLC7A11, GPX4, ACSL4, TFRC and lipid-peroxidation markers do not necessarily report the same biological state. A useful clinical assay should integrate pathway activity with functional ferroptosis readiness and should distinguish baseline prognosis from treatment-induced response.

The fourth barrier is safety. YAP/TAZ–TEAD signaling is not tumor restricted but contributes to tissue homeostasis, regeneration and repair in multiple normal organs. Sustained systemic inhibition of this pathway may therefore impair physiological maintenance and the response to tissue injury. Conversely, ferroptosis is increasingly recognized as a pathogenic mechanism in normal-tissue injury, particularly in the kidney, liver, heart and other metabolically active organs. Combining YAP/TAZ inhibition with ferroptosis-inducing therapy could consequently narrow the therapeutic window by simultaneously weakening regenerative or stress-adaptive programs and increasing iron-dependent lipid-peroxidation pressure in non-malignant tissues. This concern is particularly relevant to renal toxicity, as emerging preclinical and early clinical experience with TEAD-directed inhibitors has identified proteinuria, albuminuria and injury to renal epithelial or podocyte compartments as potential adverse effects. Importantly, whether YAP/TAZ inhibition and ferroptosis induction produce additive or synergistic toxicity in normal tissues has not yet been systematically established. Clinical development should therefore prioritize tumor-selective delivery, biomarker-guided patient selection, intermittent or sequential dosing when appropriate, and prospective monitoring of renal, hepatic and other organ-specific toxicities together with pharmacodynamic markers of ferroptosis. These safety questions should be addressed in normal-tissue organoids, orthotopic and immunocompetent models before broad systemic combination strategies are advanced into clinical trials (101–103). The fifth barrier is the lack of prospective patient-stratified trials. Current translational evidence is dominated by cell lines, xenografts, retrospective samples and engineered delivery systems, so clinical development should prioritize patient-derived organoids, orthotopic models, spatially resolved biomarkers and adaptive combination studies.

A further translational limitation concerns natural-product-based ferroptosis modulators. Although their structural diversity and multi-target pharmacology may provide opportunities for combination therapy and drug discovery, most evidence linking natural compounds to the Hippo–ferroptosis axis remains preclinical. Their apparent mechanistic breadth may also complicate identification of the pharmacologically dominant target and direct comparison with more target-defined synthetic agents. In addition, variable bioavailability, metabolic stability, formulation, pharmacokinetics, batch consistency and potential drug–drug interactions remain important barriers to clinical development. Thus, the therapeutic advantages of natural compounds over synthetic ferroptosis modulators have not yet been demonstrated in comparative clinical studies, and rigorous pharmacokinetic, toxicological and prospective efficacy studies will be required before such compounds can be considered clinically competitive (104, 105).

## 5 Conclusion and outlook

The Hippo pathway is a central regulator of cancer ferroptosis because it integrates tissue organization with iron, lipid and redox metabolism. The evidence does not support a universal statement that YAP or TAZ activation either promotes or suppresses ferroptosis. Instead, Hippo activity determines which downstream program is available: lipid-peroxidation and iron-loading programs can create vulnerability, whereas cystine uptake, glutathione production, Nrf2 activation and lipid storage can create resistance.

Four mechanistic principles emerge. First, cell contact, density, ECM and mechanical stress set the upstream threshold for ferroptosis through NF2/Merlin and YAP/TAZ activity. Second, YAP/TAZ–TEAD transcription directly controls ferroptosis effectors such as SKP2, TFRC, ACSL4, SLC7A11 and GPX4-related defense. Third, Hippo signaling is embedded in metabolic networks involving glutamine, glycolysis, lipid droplets, GCH1, Nrf2 and RNA metabolism. Fourth, epigenetic, post-translational, non-coding and stromal signals can remodel this axis and generate non-cell-autonomous resistance.

The translational opportunity is therefore not simply to inhibit YAP or induce ferroptosis in all tumors. It is to identify a tumor state in which a specific Hippo output creates a predictable ferroptosis vulnerability, then pair pathway modulation with an appropriate treatment pressure. Biomarker development should combine YAP/TAZ activity with ferroptosis execution and defense markers, while therapeutic studies should measure pharmacodynamic changes rather than relying only on tumor volume.

Several priorities should guide the next phase of research. Spatial and single-cell approaches should resolve how Hippo states distribute across tumor, stromal and immune compartments. Patient-derived organoids and orthotopic models should test whether cell-density and matrix-dependent effects persist in tissue structure. Treatment studies should use rescue experiments and dynamic measurements of iron, lipid peroxidation and antioxidant capacity to separate causal ferroptosis from nonspecific oxidative injury. Finally, clinical trials should stratify patients by combined Hippo–ferroptosis signatures and evaluate rational combinations with targeted therapy, immune checkpoint blockade or tumor-selective delivery. Such studies can translate the Hippo–ferroptosis signaling axis into clinically applicable strategies for precision ferroptosis therapy.
